# A network analysis of risk and protective factors for body image in young adult women

**DOI:** 10.1007/s40519-026-01815-x

**Published:** 2026-01-27

**Authors:** Quentin Hallez, Claire El-Jor, Rebecca Shankland

**Affiliations:** 1 Université Lumière Lyon 2, Institut de Psychologie, Laboratoire DIPHE, Bron, France; 2 Institut Universitaire de France, Paris, France; 3 Service universitaire d′addictologie de Lyon SUAL, CH le Vinatier, 69500 Bron, France

**Keywords:** Body image, Body dissatisfaction, Network analysis, Risk factors, Protective factors, Physical appearance related teasing scale validation

## Abstract

This study employed a network analysis approach to model the complex interplay of risk and protective factors for body image dissatisfaction in young French women, with the objective of mapping the psychological system connecting these variables and identifying the most central factors. A sample of 233 female students completed an online questionnaire assessing 11 constructs, including risk factors like perfectionism, thin-ideal internalization, appearance comparison, and weight stigma, alongside protective factors such as self-compassion, intuitive eating, and body appreciation. This study also presents the first psychometric validation of the Physical Appearance Related Teasing Scale (PARTS) in the French language. A Gaussian Graphical Model (GGM) network analysis revealed that body dissatisfaction (BSQ-8C) has the highest strength and betweenness centrality, confirming its role as the core hub in the model’s architecture and underscoring the relevance of the chosen variables for this study. The network showed strong direct positive links to body dissatisfaction from weight stigma (WSSQ) and appearance comparison (PACS-5), and strong negative links from the protective factors of body appreciation (BAS-2) and intuitive eating (IES-2). Weight-related teasing (PARTS) was established as a significant secondary risk factor through its robust connection with weight stigma. Sociocultural pressures (SATAQ-3) were identified as a critical bridging node, while variables such as self-compassion, social media use, and perfectionism occupied peripheral positions. This research advocates for a targeted, multi-component approach that actively works to dismantle the pillars of weight stigma and comparison while simultaneously building the distinct foundations of body appreciation and intuitive eating.

**Level of evidence; Level V, descriptive studies**

Our manuscript describes a cross-sectional design that uses a network analysis approach to map the existing correlations between variables. As this methodology is a descriptive study and does not involve an intervention (ruling out Levels I & II) or a longitudinal/case–control design (ruling out Level III), it aligns with the journal’s criteria for Level V.

**Supplementary Information:**

The online version contains supplementary material available at 10.1007/s40519-026-01815-x.

## Introduction

In an era saturated by idealized images, the relationship individuals have with their own bodies has become an important factor influencing mental well-being [1]. This complex landscape thus necessitates a detailed examination of the specific factors that shape body image [2, 3]. To establish a clear framework, it is important to define the core concept of body image. According to Grogan [4], body image can be defined as “an individual’s perceptions, thoughts, and feelings about his or her body”. When these cognitions are negative, they often result in body dissatisfaction. Grogan [4] specifies that body dissatisfaction involves one’s negative thoughts and feelings about their body, often characterized by a perceived discrepancy between their current body and their ideal body.

However, the field has evolved beyond a sole focus on pathology. Moving toward a more holistic view, scholars like Tylka and Wood-Barcalow [5] have championed the study of positive body image, which involves an overarching love and respect for the body, appreciating its functionality and health, and accepting its unique characteristics, irrespective of its alignment with societal ideals. Therefore, body image can be conceived on a continuum, ranging from body dissatisfaction to positive body image. The literature has identified numerous risk factors detrimental to body image, including individual traits like perfectionism, sociocultural pressures such as thin-ideal internalization [6] and social comparison [7], and interpersonal experiences like weight stigma [8]. Conversely, a growing body of research highlights protective factors known to foster positive body image, such as self-compassion [9], intuitive eating [10, 11], and body appreciation [12]. While the links between these individual factors and body image are established, a critical gap remains in understanding their complex systemic interplay. As such, analyzing these dynamics is crucial for developing targeted interventions and prevention strategies to promote a positive body image.

Recent network-analytic approaches have further transformed this landscape by mapping the complex, reciprocal associations among symptoms and protective mechanisms in body image and eating-disorder research. For instance, Cerea et al. [13] demonstrated, in a large community sample, that positive constructs such as body and functionality appreciation and intuitive eating occupy central and protective positions within eating-disorder symptom networks. Similarly, Meneguzzo et al. [14] integrated body image flexibility—the capacity to accept and adapt to body-related thoughts and emotions without avoidance—within a network comparing women with and without eating disorders. Their findings showed that, in clinical samples, weight and shape concerns remain central, whereas in non-clinical populations, embodiment and body-image flexibility act as crucial bridge nodes linking body concerns with adaptive self-regulation. Complementing these findings, a 2023 network study among perimenopausal women [15] confirmed that weight- and shape-related dissatisfaction persists as the most central nodes across the lifespan. Collectively, these recent studies underline that body dissatisfaction remains the structural core of body-image systems, while flexible and accepting attitudes toward one’s body constitute key protective pathways.

To operationalize this network approach, it is essential to define the specific constructs that constitute this psychological system. The literature suggests this system is composed of two primary clusters: risk factors that exacerbate dissatisfaction and protective factors that build resilience. At a dispositional level, perfectionism represents a key vulnerability for body image disturbance, particularly its socially prescribed form—the belief that one must meet unrealistically high standards to gain approval from others. This dimension is strongly correlated with body dissatisfaction, as individuals striving for an “ideal” body often experience chronic self-criticism [16]. Such internal tendencies are reinforced by sociocultural pressures, especially the promotion and internalization of the thin-ideal—the culturally dominant notion that thinness equals beauty [6]. When individuals adopt this ideal as a personal standard, perceived discrepancies between their real and ideal body become a central source of dissatisfaction.

This internalization is largely driven by appearance-based social comparison, the tendency to evaluate one’s attractiveness relative to others. A meta-analysis by Myers and Crowther [7] showed that upward comparisons—against those perceived as more attractive—consistently predict greater body dissatisfaction. With the rise of image-focused social media, these comparisons have intensified, amplifying exposure to highly curated images and fostering a continuous sense of inadequacy [17].

In addition to media influences, interpersonal experiences such as weight stigma also shape body image. Weight stigma refers to the social devaluation of individuals due to body weight and is closely associated with increased body dissatisfaction and psychological distress [8]. A particularly damaging form is weight-based teasing, especially during adolescence, which has been shown to predict long-term body dissatisfaction and disordered eating [18]. Repeated external evaluations can also lead to self-objectification, whereby individuals view their bodies from an observer’s perspective, increasing body shame and appearance anxiety [19].

Conversely, several protective factors have been identified. One powerful internal resource is self-compassion, defined as extending kindness and understanding to oneself during times of inadequacy [9]. Rather than harsh self-criticism, self-compassion promotes a balanced, caring attitude that mitigates the negative effects of perfectionism and social comparison. Another adaptive factor, intuitive eating, involves trusting internal hunger and satiety cues while rejecting restrictive “diet mentality” rules [10, 11]. This flexible eating style reduces food-related anxiety and fosters a healthier, more trusting relationship with the body. Finally, body appreciation—valuing the body for its functionality and health rather than its appearance—has emerged as a core component of positive body image [12]. Cultivating body appreciation builds self-worth that is less dependent on external standards, buffering against sociocultural and interpersonal pressures.

While body image concerns affect individuals across the gender spectrum, the present study focuses specifically on a sample of young women. This decision is informed by several considerations grounded in the literature. Firstly, extensive research consistently reveals a significant gender disparity in body image, with young women reporting particularly high rates of body dissatisfaction in comparison to young men [20]. Furthermore, studies investigating sociocultural pressures [2, 3] indicate this demographic is disproportionately exposed to influences from media, peers, and family that promote a narrow thin-ideal. Secondly, to ensure methodological coherence, this study uses a homogeneous sample (age and gender) to more clearly examine the relationships between the chosen variables. Therefore, the findings of this network analysis are intended to illuminate the specific dynamics within this particular population. Focusing on young women is especially relevant, as extensive research has identified them as one of the demographics most at risk for developing body image disturbances [1, 21, 22].

Building on this framework, the present study aimed at applying a network analysis approach to model the complex interplay between these risk and protective factors within a sample of young women. The primary objective was to identify which factors emerge as the most central in this network, potentially highlighting key leverage points for future clinical interventions. A secondary methodological objective was to conduct the first psychometric validation of the Physical Appearance Related Teasing Scale (PARTS) in French.

## Method

### Participants

Participants were recruited from [University name removed for blind review]. Recruitment was conducted via announcements during undergraduate courses. Interested individuals received a link to an online Lime Survey® questionnaire. Inclusion criteria were: (1) identifying as female, (2) current student status, and (3) being 18–30 years old. All participants provided written informed consent and received no compensation for participation.

A total of 326 individuals accessed the online survey; 71 individuals exited after providing consent, and 22 provided partial data. These 93 incomplete responses were excluded from the final analysis. Thus, the final sample comprised 233 participants (*M*age = 20.7, SD = 2.4). While there are no absolute rules for sample size in network analysis, recent guidelines emphasize the importance of having a sample large enough to ensure the stability of the estimated network parameters (i.e., edge weights and centrality indices). With 233 participants, our sample size is considered adequate for estimating a stable network, particularly for networks with a moderate number of nodes [23]. The study was conducted in accordance with the Declaration of Helsinki and received ethical approval from the University of Nantes ethics committee.

### Material and procedure

Data were collected using an online questionnaire designed on the Lime Survey® platform. The questionnaire consisted of eleven distinct self-report scales. To control for potential order effects, the presentation of these scales was randomized for each participant. After completing the scales, participants were asked to answer demographic questions about their gender, age, and student status.

### Measures

*Body Dissatisfaction* was assessed using the 8-item French version [24] of the Body Shape Questionnaire (BSQ-8C) [25], a 6-point Likert-type questionnaire (1 = "Never", 6 = "Always"). The BSQ-8C demonstrated excellent internal consistency (Cronbach’s *α* = 0.92; McDonald’s *ω* = 0.92).

*Perfectionism* was assessed using the first part of the French Perfectionism Questionnaire validated by [26], consisting of 7 items (1 = "Does not describe me at all", 5 = "Describes me perfectly"). This subscale demonstrated good internal consistency (Cronbach’s *α* = 0.82; McDonald’s *ω* = 0.81).

*Thin-Ideal Internalization* was evaluated using a modified and abbreviated version of the Sociocultural Attitudes Towards Appearance Questionnaire 3 (SATAQ-3) [27]. While the original is composed of 30 items, this version includes 18. Some similar items were removed, and those exclusively concerning magazines and advertisements were replaced with items about social networks. This questionnaire uses a 5-point Likert scale, ranging from 1 ("Definitely Disagree") to 5 ("Definitely Agree"). This adapted scale demonstrated excellent internal consistency (Cronbach’s *α* = 0.90; McDonald’s *ω* = 0.90). See Supplement for results of confirmatory factor analysis.

*Appearance Comparison* was measured with a French version of the Physical Appearance Comparison Scale (PACS-5) [28], a 5-point Likert scale (1 = “Never”, 5 = “Always”). The internal consistency for this scale was good (Cronbach’s *α* = 0.78; McDonald’s *ω* = 0.81).

*Weight Stigma* was evaluated using the French validation [29] of the 12-item Weight Self-Stigma Questionnaire (WSSQ) [30], a 5-point Likert scale (1 = “Completely Disagree”, 5 = “Entirely Agree”). It demonstrated good internal consistency (Cronbach’s *α* = 0.87; McDonald’s *ω* = 0.87).

*Weight-Related Teasing* was assessed using the Physical Appearance Related Teasing Scale (PARTS) [31] translated into French following recommended procedures [32]. Of the 18 original items, only the 11 directly related to body image were retained. Participants responded using a Likert scale from 1 (“Never”) to 5 (“Frequently”). As the French version’s factorial structure and psychometrics needed confirmation, these analyses appear in the Results section.

*Self-Objectification* was measured using the Self-Objectification Questionnaire (SOQ) [33]. It includes 10 items, evaluated on a Likert scale ranging from 1 ("Low Impact") to 11 ("High Impact"). This scale demonstrated good internal consistency (Cronbach’s *α* = 0.73; McDonald’s *ω* = 0.73).

*Social Media Use* was assessed by an adapted version of the Media and Technology Usage and Attitudes Scale (MTUAS) [34]. This tool measures the frequency of use of certain social networks as well as attitudes towards them. Original items referring to Facebook were adapted for Instagram and TikTok. The version used includes 9 items, to which participants respond on a Likert scale from "Never" (1) to "All the Time" (10). This scale also demonstrated good internal consistency (Cronbach’s *α* = 0.78; McDonald’s *ω* = 0.78).

*Self-Compassion* was evaluated using the brief 12-item version [35] of the Self-Compassion Scale (SCS) derived from the French translation of the SCS-26 [36]. Participants respond using a Likert scale from "Almost Never" (1) to "Almost Always" (5). The scale’s internal consistency was modest (Cronbach’s *α* = 0.66; McDonald’s *ω* = 0.66). This value is below the conventional 0.70 threshold for acceptable reliability [37]. With McDonald’s *ω* (*ω* = 0.66) providing a nearly identical estimate, this was accepted as a limitation of this abbreviated scale. Its potential to attenuate the node’s true connections is discussed in detail in the Discussion and should be considered when interpreting related findings.

*Intuitive Eating* was measured with a French version [38] of the Intuitive Eating Scale-2 (IES-2) [10, 11]. Participants responded to 23 items using a 5-point Likert scale, from "Strongly disagree" (1) to "Strongly agree" (5). The total score was used, which demonstrated really good internal consistency (Cronbach’s *α* = 0.88; McDonald’s *ω* = 0.88).

*Body Appreciation* was assessed using a French version of the Body Appreciation Scale 2 (BAS-2) [5], a 10-item scale responded to by a Likert scale (1 = "Never”, 5 = "Always"). This scale demonstrated excellent internal consistency (Cronbach’s *α* = 0.95; McDonald’s *ω* = 0.95).

### Statistical analyses

Prior to the analyses, data quality was assessed. As detailed in the Participants section, only complete responses were retained for the final sample (*N* = 233). Furthermore, the final dataset was visually inspected for nonsensical response patterns (e.g., invariant "straight-line" responding across all items). While no specific embedded attention-check items were used, no problematic response patterns were identified in the final sample.

All statistical analyses were conducted using JASP software (0.19.20). Prior to these analyses, a confirmatory factor analysis (CFA) was conducted to validate the unidimensional structure of the PARTS scale translated into French. The CFA model identification was achieved by fixing the factor loading of the first item to 1.0, which is the standard default procedure.

Subsequently, a Pearson’s correlation was performed among the different scales to examine the links between the measured variables. This was followed by a network analysis examining the relationships between the variables of interest. We estimated a Gaussian Graphical Model (GGM), wherein the network’s connections, or "edges", represent partial correlation coefficients between nodes [23]. Given that the data were derived from Likert-type scales, the GGM framework is particularly appropriate as it accommodates non-equidistant ordinal data through the estimation of polychoric correlations [39].

To produce a parsimonious and interpretable network, we implemented regularization. Specifically, we applied the least absolute shrinkage and selection operator (LASSO) algorithm, which effectively prunes the network by shrinking spurious edges to zero [40]. The LASSO tuning parameter (γ) was set to 0.5, a value selected to minimize the Extended Bayesian Information Criterion (EBIC). This process yields a ‘weighted’ adjacency matrix, enhancing the interpretability and reliability of the final network structure [23].

The resulting network structure was analyzed by examining both the weighted adjacency matrix and a series of centrality indices [23]. The weighted matrix provides a measure of the strength of the relationships between nodes, with higher weights indicating stronger partial correlations [40]. To identify the relative importance of each node within the network, four centrality indices were calculated: Strength, Closeness, Betweenness, and Expected Influence. Strength is a measure of a node’s direct influence in the network, calculated as the sum of its non-zero edge weights [23]. Closeness represents the inverse sum of the shortest paths from a node to all other nodes, while Betweenness reflects the number of times a node lies on the shortest path between two other nodes [40]. Finally, Expected Influence provides a comprehensive measure of a node’s overall influence by aggregating its edge weights, taking into account both positive (excitatory) and negative (inhibitory) relationships [41].

To assess the reliability and robustness of the estimated network, the stability of its parameters was tested using two recommended non-parametric bootstrap approaches (*n* = 2000). First, the accuracy of the edge weights was assessed by constructing 95% confidence intervals around each estimated connection. Narrow confidence intervals indicate that the strength of network connections is estimated reliably and precisely. Second, the stability of the centrality indices was verified using a case-dropping bootstrap procedure. This involves recalculating the indices after progressively removing participants from the sample to ensure that the relative importance of the nodes remains constant and interpretable.

## Results

### French validation of the PARTS scale

Based on a preliminary analysis of a full 11-item model, a decision was made to re-specify the model by removing the 7th item (i.e., “Did your father ever make jokes that referred to your weight?”). This item was targeted for removal due to its poor individual measure of sampling adequacy (*MSA* = 0.47) and weak factor loading (*λ* = 0.29).

The revised 10-item unidimensional model was then evaluated. Given that several items exhibited high skewness and kurtosis (values > 3), indicating a violation of the multivariate normality assumption, we employed the robust weighted least squares mean and variance adjusted (WLSMV) estimator. The data’s suitability for factor analysis was excellent; the overall Kaiser–Meyer–Olkin measure was 0.83, with all individual item *MSA* values exceeding the recommended 0.50 threshold. Bartlett’s test of sphericity remained significant, *χ*^2^(45) = 2599.54, *p* < 0.001.

The CFA yielded mixed fit indices. While the incremental fit indices were excellent (CFI = 0.99, TLI = 0.99) and the standardized root mean square residual (SRMR) was good (SRMR = 0.078), the root mean square error of approximation (RMSEA) was high (RMSEA = 0.102, 90% CI [0.08, 0.12]). Despite the elevated RMSEA, all retained items loaded significantly and substantially onto the single latent factor (all *ps* < 0.001), as detailed in Table 1.

**Table 1:** Factor loadings paired with the confirmatory factor analysis

Items	Factor loadings (standardized)
When you were a child, did you feel that your peers were staring at you because you were overweight? *Lorsque vous étiez enfant, avez-vous senti que vos pairs vous regardaient fixement parce que vous étiez en surpoids ?*	0.87
When you were a child, did you ever feel like people were making fun of you because of your weight? *Lorsque vous étiez enfant, avez-vous senti que les gens se moquaient de vous à cause de votre poids ?*	0.90
Were you ridiculed as a child about being overweight? *Avez-vous été ridiculisée dans votre enfance à propos de votre surpoids ?*	0.98
When you were a child, did people make jokes about you being too big? *Lorsque vous étiez enfant, les gens se moquaient-ils de vous parce que vous étiez trop grosse ?*	0.97
When you were a child, were you laughed at for trying out for sports because you were heavy? *Lorsque vous étiez enfant, vous est-il arrivé que l’on se moque de vous parce que vous vouliez faire du sport alors que vous étiez lourde ?*	0.82
Did your brother(s) or other male relatives call you names like "fatso" when they got angry at you? *Est-ce que votre (vos) frère(s) ou d’autres membres masculins de votre famille vous ont appelée par des noms comme « la grosse» lorsqu’ils étaient en colère contre vous ?*	0.55
Did other kids call you derogatory names that related to your size or weight? *Est-ce que d’autres enfants vous ont appelée par des noms dénigrants liés à votre poids?*	0.79
Did you ever feel like people were pointing at you because of your size or weight? *Est-ce que vous avez déjà senti que les gens vous désignaient du doigt à cause de votre poids ?*	0.80
Were you the brunt of family jokes because of your weight? *Avez-vous fait l’objet de blagues en famille à cause de votre poids ?*	0.42
Did people point you out of a crowd because of your weight? *Est-ce que des personnes vous ont désignée du doigt dans une foule à cause de votre poids ?*	0.77

All factor loadings are significant at *p* < 0.001

### Bivariate correlations

Table 2 shows the descriptive statistics associated with each variable. The Pearson’s correlation matrix, shown in Table 3, revealed that 27 of the 55 possible correlations between the study variables were statistically significant.

**Table 2:** Descriptive statistics for all study variables

Variables	M	SD	Skewness	Kurtosis	[Min; Max]
Body dissatisfaction (BSQ-8C)	24.07	9.98	0.36	−0.85	[8; 48]
Perfectionism (QPR)	24.91	5.43	−0.34	−0.25	[7; 35]
Sociocultural pressures (SATAQ-3)	51.62	13.71	−0.10	−0.47	[18; 82]
Appearance comparison (PACS)	13.01	0.16	−0.16	−0.56	[4; 20]
Weight self-stigma (WSSQ)	30.59	9.18	0.01	−0.5	[12; 56]
Perfectionism (PARTS-R)	17.89	8.13	1.46	1.53	[11; 49]
Self-objectification (LSOQ)	74.44	14.00	−0.36	0.06	[25; 107]
Social media use (MTUAS)	38.09	14.00	−0.98	1.47	[0; 70]
Self-compassion (SCS)	31.67	4.61	0.04	−0.22	[19; 45]
Intuitive eating (IE)	68.67	15.11	−0.08	−0.60	[30; 103]
Body appreciation (BAS)	33.29	8.74	−0.18	−0.35	[10; 50]

**Table 3:** Pearson’s correlations among body image risk and protective factors

Variable		1	2	3	4	5	6	7	8	9	10
1. BSQ-8C		–									
2. QP		0.08	–								
3. SATAQ-3		0.45***	0.01	–							
4. PACS-5		0.59***	−0.00	0.50***	–						
5. WSSQ		0.70***	−0.04	0.44***	0.44***	–					
6. PARTS		0.36***	−0.09	0.21**	0.24***	0.46***	–				
7. LSOQ		0.18**	0.12	0.18**	0.13*	0.12	0.08	–			
8. MTUAS		0.09	−0.05	0.14*	0.14*	0.03	0.06	0.04	–		
9. SCS		−0.07	0.03	−0.10	−0.15*	−0.00	−0.07	−0.00	−0.05	–	
10. IES-2		−0.70***	0.01	−0.34***	−0.45***	−0.60***	−0.30***	−0.06	−0.04	0.23***	–
11. BAS-2		−0.72***	−0.03	−0.33***	−0.51***	−0.59***	−0.21***	−0.03	−0.06	0.30***	0.60***

*p < 0.05; **p < 0.01; ***p < 0.001. *BSQ-8C* Body Shape Questionnaire; QP Questionnaire of Perfectionism, *SATAQ-3* Sociocultural Attitudes Towards Appearance Questionnaire, *PACS-5* Physical Appearance Comparison Scale, *WSSQ* Weight Self-Stigma Questionnaire, *PARTS* Physical Appearance Related Teasing Scale, *LSOQ* Likert Self-Objectification Questionnaire, *MTUAS* Media and Technology Usage and Attitudes Scale, *SCS* Self-Compassion Scale, *IES-2* Intuitive Eating Scale-2, *BAS-2* Body Appreciation Scale 2. Thresholds for correlation strength are defined as weak (*r* [0.10–0.29], moderate (*r* [30–0.49]), and strong (*r* > 0.50), according to Cohen [42]

### Network analysis

**Fig. 1:**
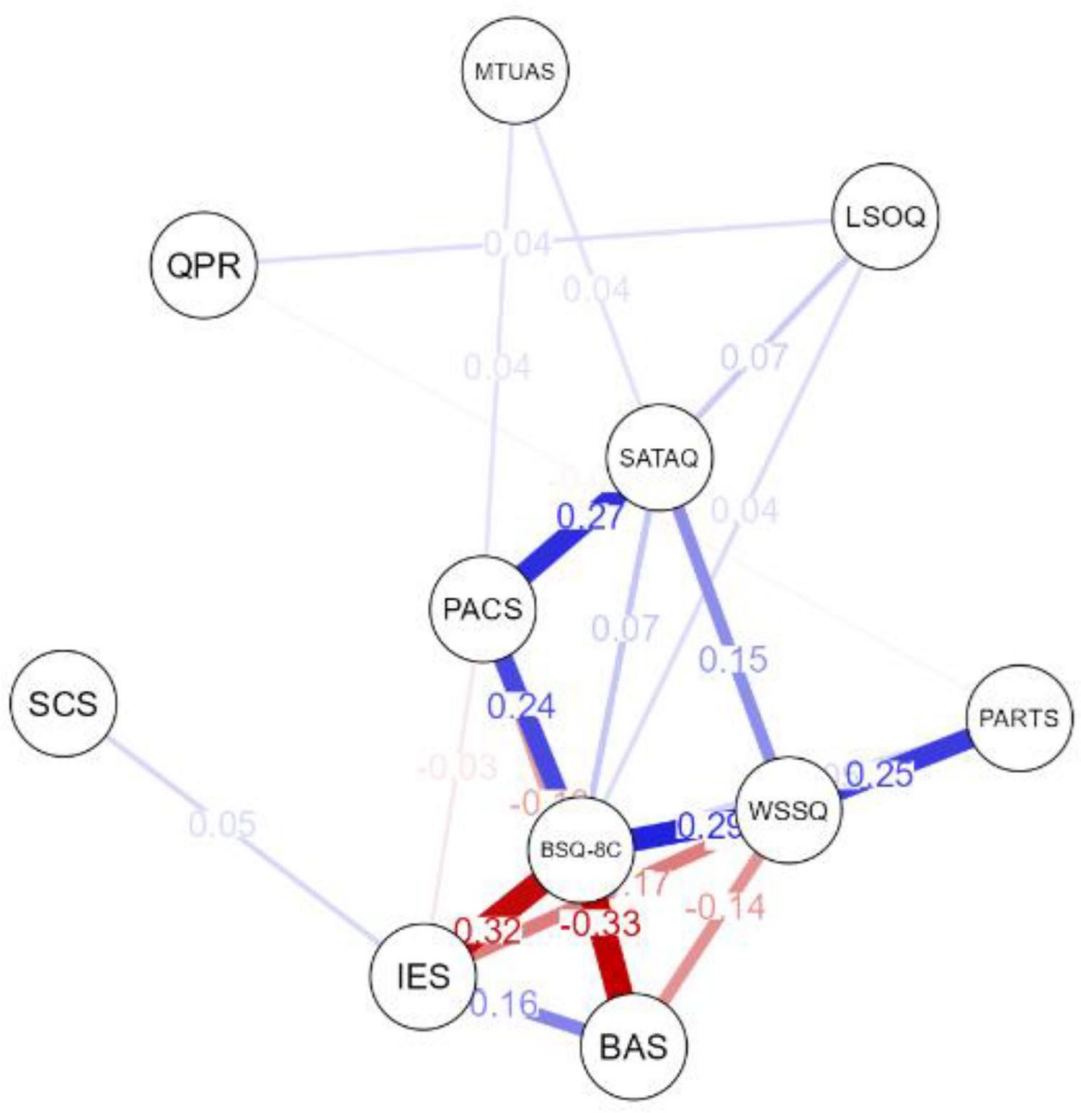
Network analysis of risk and protective factors for body dissatisfaction. *Note.* Network of psychological variables estimated using the EBICglasso (Extended Bayesian Information Criterion graphical LASSO) model, based on regularized partial correlations. Each node represents a measured psychological variable: *BSQ-8C* Body Dissatisfaction, *BAS* Body Appreciation, *IES* Intuitive Eating, *WSSQ* Weight Stigma, *PACS* Appearance Comparison; *SATAQ* Thin-Ideal Internalization, *PARTS* Weight-related Teasing, *SCS* Self-Compassion, *QPR* Perfectionism, *LSOQ* Self-Objectification, *MTUAS* Social Media Use. The lines connecting the nodes are "edges", representing the partial correlations. Blue edges denote positive relationships, and red edges denote negative relationships. The thickness and saturation of an edge are proportional to the strength of the correlation. Nodes with more and stronger connections are more central to the network’s structure

A visual inspection of the network graph, presented in Fig. 1, reveals a clear core–periphery structure. At the center of this structure is body dissatisfaction (BSQ-8C), which acts as the primary hub of the network. Notably, BSQ-8C is strongly and negatively linked (indicated by thick red edges) to intuitive eating (IES) and body appreciation (BAS), visually supporting the interpretation of these variables as protective factors. Conversely, it displays strong positive links (blue edges) with appearance comparison (PACS) and weight stigma (WSSQ), highlighting them as prominent risk factors. Furthermore, while more distant from the main hub, the internalization of ideals (SATAQ) appears to hold a pivotal position in the model, primarily through its robust connection to PACS.

In contrast, other variables such as self-compassion (SCS), perfectionism (QPR), self-objectification (LSOQ), and social media use (MTUAS) are situated on the network’s periphery, suggesting they play a more secondary or annex role in this model.

**Fig. 2:**
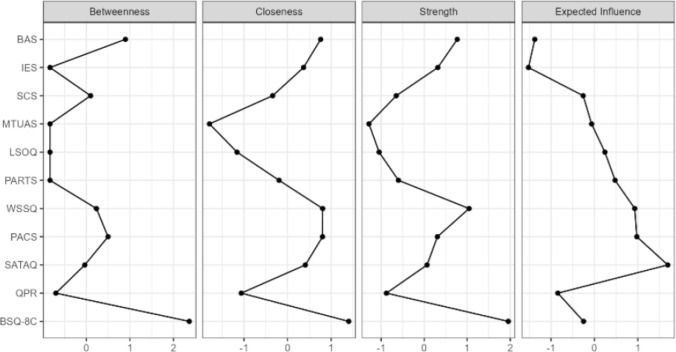
Centrality plot for network analysis paired with the network analysis

The centrality indices, visible in Fig. 2, were used to identify the most structurally important nodes within the network. Body issatisfaction was identified as the most central and influential node. It exhibited the highest Strength centrality, indicating the strongest overall degree of connection to other nodes. It also possessed the highest Betweenness centrality, positioning it as the primary intermediary pathway connecting disparate parts of the network. Furthermore, its high positive Expected Influence score suggests it functions as a key activating node within this system. This finding confirms its role as a core hub in the model’s architecture, underscoring the relevance of the chosen variables for this study.

Two variables emerged as primary protective factors, characterized by strong negative connections to BSQ-8C and high centrality. BAS and IES demonstrated the strongest negative edge weights in the entire network in their connections with BSQ-8C (edge weights = −0.32 and −0.31, respectively). Their protective role is confirmed by their centrality scores (Fig. 2): both possess high Strength and the most negative Expected Influence, indicating a strong deactivating function on the network. Their high Closeness centrality further suggests they can efficiently counteract distress across the system. A third variable, SCS, showed a weaker protective function. It is not directly connected to BSQ-8C but has a weak positive link to BAS (edge weight = 0.13), suggesting an indirect protective pathway. Its low centrality scores, however, indicate a less influential role compared to BAS and IES.

A set of highly interconnected variables function as risk factors, all displaying positive Expected Influence. PACS and WSSQ function as primary risk factors. Both are directly and positively connected to BSQ-8C (edge weights = 0.23 and 0.28, respectively) and exhibit high Strength centrality. Their positive Expected Influence confirms their activating role. Two other variables act as significant secondary risk factors through their strong links with the primary ones. SATAQ is strongly linked to PACS (edge weight = 0.26), forming a key pathway related to appearance ideals. PARTS is strongly linked to WSSQ (edge weight = 0.24), forming another pathway related to weight-based experiences. While SATAQ and PARTS have lower overall centrality, their specific, strong connections make them important components of the risk-related structure.

Conversely, nodes with the lowest centrality scores across all indices, such as MTUAS, LSOQ, and QPR, were located on the network’s periphery. This is visually represented by their weak or absent edges, signifying minimal direct relationships with other variables after controlling for the rest of the network.

### Network stability and accuracy

**Fig. 3:**
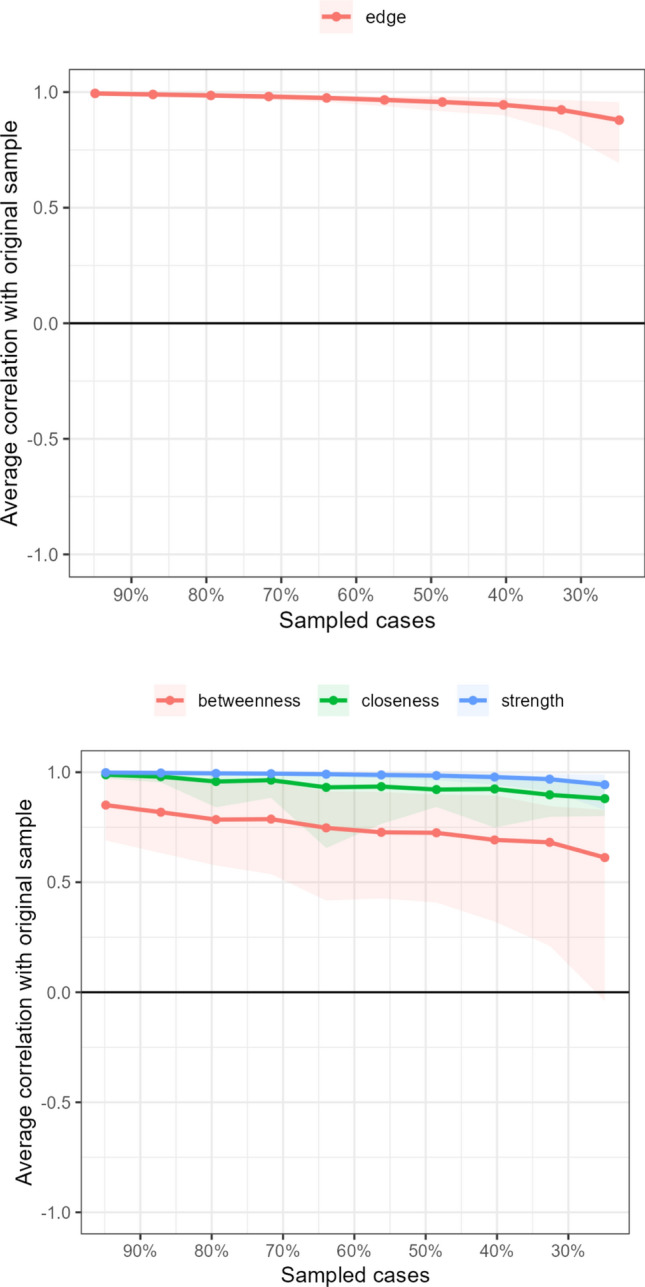
Stability analysis of network edge weights (*top panel*) and centrality indices (*bottom panel*) using a case-dropping bootstrap procedure

To evaluate the reliability and robustness of the estimated network, two stability analyses were conducted using a case-dropping bootstrap procedure (2000 bootstraps). The results, depicted in Fig. 3, assess the stability of both the edge weights and the centrality indices.

First, the accuracy of the edge weights was examined (Fig. 3, top panel). The analysis revealed excellent stability for the network’s structure. The average correlation between the edge weights of the subsampled networks and the original network remained exceptionally high, staying above 0.95 even when up to 70% of the cases were removed from the original sample. This indicates that the estimated edge weights are highly robust and not dependent on a specific subset of participants, providing confidence in the overall network structure.

Second, the stability of the centrality indices was assessed (Fig. 3, bottom panel). Strength and Closeness demonstrated excellent stability, with average correlations remaining at or near 1.0 across all levels of subsampling. This suggests that the identification of the most central nodes based on these metrics is highly reliable. Betweenness was found to be less stable, a common finding for this metric, attributable to the fact that Betweenness is a global network metric that can be sensitive to small fluctuations in the network structure [23]. By examining the lower bound of this confidence interval, we can determine the correlation stability (CS) coefficient. This lower bound drops below a correlation of 0.7 when approximately 60% of the sample is removed (at the 40% "Sampled cases" mark). This yields a CS-coefficient for Betweenness of approximately 0.4. According to established guidelines, a CS-coefficient above 0.25 is considered acceptable, and a value above 0.5 is preferred [23]. As our result of 0.4 is comfortably above the minimum threshold, the Betweenness centrality metric can be interpreted with sufficient confidence.

## Discussion

Moving beyond traditional linear models that have often examined factors influencing body image in isolation, the present study employed a network analysis approach to map the complex, systemic interplay among established risk and protective factors in young women. By conceptualizing these variables as an interconnected system, this research aimed to identify not only which factors are related to body dissatisfaction but also to reveal the underlying structure of their relationships and their respective roles within the network. Beyond its theoretical contributions, this study conducted the initial validation of the French version of the Physical Appearance Related Teasing Scale (PARTS). The scale demonstrated promising, though mixed, psychometric properties. While its structural fit (as indicated by the high RMSEA in our CFA) warrants further investigation, its construct validity was well-supported.

First and foremost, the analysis provides a critical validation of the study’s conceptual framework. As hypothesized, body dissatisfaction emerged as the undisputed central node of the network, exhibiting the highest scores across centrality indices. This structural prominence confirms that the selected variables form a coherent psychological system organized around body dissatisfaction. Regarding its high betweenness, this suggests that body dissatisfaction is structurally connecting and channeling the influence of other variables within the system. This finding provides a robust foundation for interpreting the specific pathways discussed below.

Consistent with the literature, body dissatisfaction was strongly and negatively associated with body appreciation and intuitive eating. The particularly strong negative edges between body dissatisfaction and these two variables (edge weights = −0.32 and −0.31, respectively) are noteworthy. This suggests that fostering an attitude of appreciation for one’s body [5] and attunement to internal hunger/satiety cues may be powerful buffers against body dissatisfaction. Interestingly, the final network model revealed no direct edge between body appreciation and intuitive eating. This suggests they may function as two distinct protective pathways. While often correlated in bivariate analyses, their conditional independence in this network implies that interventions could target each separately [43].

The analysis also confirmed and clarified the potent role of several risk factors. Weight stigma and appearance comparison emerged as having the strongest direct links to bodydissatisfaction. This alignment with prior research [44, 45] is critical, as the network model demonstrates these relationships persist even when controlling for all other variables, pointing to them as primary intervention targets. This also provides strong support for the Tripartite Influence Model (TIM) [46], which posits that sociocultural pressure from peers, parents, and the media fosters body dissatisfaction through the thin-ideal internalization and appearance comparison. Consistent with the model’s predictions, the network analysis confirmed that both thin-ideal internalization and comparison are indeed significantly linked to body dissatisfaction.

However, the network analysis also offers a more nuanced view of the relationship between these two mediators. A strong, positive edge was observed directly connecting internalization and comparison. While the tripartite model often treats these as two parallel pathways, this finding raises an important question about their potential interplay. It is plausible that these mechanisms may not be entirely independent. This strong link is consistent with a model where thin-ideal internalization could act as a cognitive antecedent, creating a standard that subsequently motivates and directs social comparison. Because our cross-sectional design precludes causal inference, this finding nevertheless points to a valuable avenue for longitudinal work on the potential sequencing of these two processes. Although this specific temporal sequence remains hypothetical, our results clearly show that these risk factors constitute a tightly knit, reinforcing system. It is this structural reality of a "risk cluster" that has direct clinical implications. This interconnectedness argues strongly against isolated, single-focus interventions. To be effective, therapeutic approaches should be multi-component, designed to dismantle the entire reinforcing system rather than just one of its parts.

A compelling, yet cautionary, finding was the unexpected peripheral role of several variables commonly linked to body image, such as social media use, self-objectification, perfectionism, and self-compassion. This pattern may reflect an important methodological finding regarding generic versus domain-specific variables. The most central nodes in our network (e.g., weight stigma and appearance comparison) are specific to body dissatisfaction, whereas these peripheral nodes represent generic personality traits or broader behaviors. Our measurement choices (detailed below) likely compounded this issue by failing to capture the domain-specific aspects of these broader constructs. For instance, the social media use node measured generic frequency of use, not the domain-specific nature of content consumed (e.g., ‘fitspiration’) [47], which likely explains its weak, indirect links [48, 49]. Similarly, the absence of a strong link for perfectionism may reflect the generic, adaptive subscale chosen, rather than the maladaptive, self-critical components more robustly linked to body dissatisfaction [21, 50]. Likewise, the weak self-compassion (SCS) association supports this hypothesis, suggesting that a body-specific measure could have produced a stronger link. This theoretical explanation is further compounded by a significant scale limitation: the brief scale’s modest internal consistency, which may have artificially attenuated its true relationship strength. Finally, self-objectification is also peripheral. While it is domain-specific, its weak link is still surprising. A plausible, though speculative, interpretation is that self-objectification acts as a more stable, distal vulnerability factor (a trait), while our network captures more proximal, state-like factors like daily comparisons. This hypothesis, however, requires longitudinal testing.

### Strengths and limitations

The primary strength of this study is its novel methodological approach. By employing network analysis, this research moves beyond viewing body image factors in isolation, offering a valuable, system-level visualization of the psychological dynamics at play. A second major contribution is the initial validation of a 10-item French version of the PARTS, providing a necessary tool for future research in French-speaking populations. We note this as an initial validation, as its structural fit warrants further investigation, as detailed below. The resulting network model was also found to be highly robust, as confirmed by bootstrap analyses ensuring the stability of the edge weights and centrality indices.

However, the findings of this study should be interpreted in light of several limitations, which also point toward important avenues for future research. First, the sample was composed exclusively of young French female undergraduate students. While this demographic is known to be at high risk for body image disturbances [1, 21, 22], this homogeneity significantly restricts the generalizability of our findings. Sociocultural pressures, the internalization of specific appearance ideals, and even the experience of weight stigma are deeply embedded in cultural context. Therefore, the network structure observed may not be generalizable to young women in non-Western or other European cultural settings. Furthermore, this non-clinical student sample may not represent the network dynamics of individuals with diagnosed body image disturbances or eating disorders, limiting the study’s direct clinical applicability. It is plausible that in a clinical population, the connections between factors like perfectionism or self-objectification and the central hub of body dissatisfaction might be significantly stronger than the peripheral role observed in our model. Furthermore, we did not collect other key demographic data, such as ethnicity, socioeconomic status, or body mass index (BMI). The absence of this information, particularly BMI, which is highly relevant to body image research, further limits the specific context of our findings and their generalizability. Future research is essential to replicate and compare this network structure in both clinical samples and more culturally diverse populations.

Second, this study utilized a cross-sectional design, capturing a snapshot of the relationships between variables at a single point in time. While network analysis reveals the strength of conditional associations, it does not permit inferences about causality or the temporal precedence of these factors. For example, the strong link observed between thin-ideal internalization and appearance comparison is theoretically plausible as a sequential pathway, but this hypothesis can only be tested using longitudinal data. Future research using cross-lagged panel models or dynamic network analysis on longitudinal data would be a critical next step to understand how this system of risk and protective factors evolves and influences body image trajectories.

Third, limitations related to measurement specificity must be considered. This study conceptualized body dissatisfaction as a continuous variable. While valuable, this dimensional approach does not account for a potential clinical threshold. It is possible that the network structure could differ for individuals whose dissatisfaction reaches a clinical level of severity, a key question for future comparative studies. Furthermore, the peripheral role observed for variables like social media use and perfectionism highlights the importance of measurement choice. The scales used measured frequency of social media use and adaptive perfectionism, whereas measures of content-specific media engagement or maladaptive perfectionism may have yielded a more central role. Despite these limitations, the present study provides a clear and robust map of the psychological system surrounding body dissatisfaction.

Finally, limitations related to the psychometric validation of the PARTS scale must be considered. As per methodological recommendations, we used a robust WLSMV estimator, which revealed mixed fit indices. Specifically, a high RMSEA (0.10) was observed, despite excellent CFI, TLI, and SRMR values. This suggests the 10-item structure may require further refinement. While we proceeded with this scale due to its strong performance on other indices and its theoretical importance, future research using a formal Multitrait-Multimethod (MTMM) approach or establishing test–retest reliability would be ideal to fully confirm its psychometric properties in this population.

### What is already known on this subject?

It is well-established through traditional linear and mediation models that body dissatisfaction is influenced by a constellation of risk and protective factors. Key risk factors include the internalization of sociocultural appearance ideals, appearance-based social comparison, and interpersonal experiences like weight stigma. Conversely, positive body image constructs, particularly body appreciation and intuitive eating, are consistently identified as protective factors that buffer against body dissatisfaction [51, 52].

### What this study adds?

This study moves beyond linear models by applying a network analysis approach to map the complex systemic interplay of these risk and protective factors. The analysis clarifies primary intervention targets by showing that weight stigma and appearance comparison have the strongest direct positive links to body dissatisfaction, while body appreciation and intuitive eating have the strongest direct protective links. Crucially, this study reveals that body appreciation and intuitive eating are conditionally independent in the network, suggesting they are two distinct and powerful pathways for intervention. Alongside this primary theoretical contribution, the study provides a methodological advance by offering an initial validation of a French version of the PARTS, offering a useful tool for future research.

## Supplementary Information

Below is the link to the electronic supplementary material.

Supplementary file 1.

## Data Availability

All research data are publicly available via an anonymous Open Science Framework (OSF) link. The link can be accessed by visiting https://osf.io/vcqnd/?view_only=50973d740b7343be86e5e582b839a459
