# Supplementary material for: A network analysis of risk and protective factors for body image in young adult women
Source: Eat Weight Disord. 2026 Jan 27;31(1):15. doi: 10.1007/s40519-026-01815-x (PMC12901111; doi:10.1007/s40519-026-01815-x)
Supplement: Supplementary file 1 [file 40519_2026_1815_MOESM1_ESM.docx]

**Supplement**

A confirmatory factor analysis (CFA) was conducted to examine the factorial validity of the adapted 18-item SATAQ-3 model. Given the non-normal distribution of the data and following current methodological recommendations, the analysis was performed using the robust Weighted Least Squares Mean and Variance adjusted (WLSMV) estimator.

The hypothesized model demonstrated a mixed fit to the data. The incremental fit indices were acceptable (CFI = .94, TLI = .93), and the Standardized Root Mean Square Residual indicated a good fit (SRMR = .079). However, the Root Mean Square Error of Approximation was poor and indicated a problematic fit (RMSEA = .109, 90% CI [.099, .119]), falling just above the recommended upper threshold of .10.

Despite the elevated RMSEA, all standardized factor loadings were significant (*p* < .001) and substantial, ranging from .69 to .95.

Given that the CFI, TLI, and SRMR indices were acceptable and the factor loadings were strong, we retained this model for the main analysis. However, the poor RMSEA is noted as a significant psychometric limitation of this scale in our sample (as mentioned in the main manuscript's Limitations section).
